# Leucine-Rich Repeat Kinase 2 (Lrrk2)-Sensitive Na^+^/K^+^ ATPase Activity in Dendritic Cells

**DOI:** 10.1038/srep41117

**Published:** 2017-01-25

**Authors:** Zohreh Hosseinzadeh, Yogesh Singh, Derya R. Shimshek, Herman van der Putten, Carsten A. Wagner, Florian Lang

**Affiliations:** 1 Department of Cardiology, Vascular Medicine and Physiology, University of Tübingen, Gmelinstr. 5, D-72076 Tübingen, Germany; 2 Experimental Retinal Prosthetics Group, Institute for Ophthalmic Research, University of Tübingen, Tübingen, Germany; 3 Department of Neuroscience, Novartis Institutes for BioMedical Research, CH-4002 Basel, Switzerland; 4 National Contest for Life (NCL) Foundation, 203555 Hamburg, Germany; 5 Institute of Physiology, University of Zurich, Winterthurerstrasse 190, CH-8057 Zurich, Switzerland

## Abstract

Leucine-rich repeat kinase 2 (Lrrk2) has been implicated in the pathophysiology of Parkinson’s disease. Lrrk2 is expressed in diverse cells including neurons and dendritic cells (DCs). In DCs Lrrk2 was shown to up-regulate Na^+^/Ca^2+^-exchanger activity. The elimination of Ca^2+^ by Na^+^/Ca^2+^ -exchangers requires maintenance of the Na^+^ gradient by the Na^+^/K^+^ -ATPase. The present study thus explored whether Lrrk2 impacts on Na^+^/K^+^ -ATPase expression and function. To this end DCs were isolated from gene-targeted mice lacking Lrrk2 (*Lrrk2*^−/−^) and their wild-type littermates (*Lrrk2*^+/+^). Na^+^/K^+^ -ATPase activity was estimated from K^+^ induced, ouabain sensitive, current determined by whole cell patch clamp. Na^+^/K^+^ -ATPase α1 subunit transcript and protein levels were determined by RT-qPCR and flow cytometry. As a result, the K^+^ induced current was significantly smaller in *Lrrk2*^−/−^ than in *Lrrk2*^+/+^ DCs and was completely abolished by ouabain (100 μM) in both genotypes. The K^+^ induced, ouabain sensitive, current in *Lrrk2*^+/+^ DCs was significantly blunted by Lrrk2 inhibitor GSK2578215A (1 μM, 24 hours). The Na^+^/K^+^ -ATPase α1 subunit transcript and protein levels were significantly lower in *Lrrk2*^−/−^ than in *Lrrk2*^+/+^ DCs and significantly decreased by Lrrk2 inhibitor GSK2578215A (1 μM, 24 hours). In conclusion, Lrrk2 is a powerful regulator of Na^+^/K^+^ -ATPase expression and activity in dendritic cells.

Leucine-rich repeat kinase 2 (Lrrk2) has been implicated in the pathophysiology of Parkinson’s disease (PD)^1^,^2^,^3^. Lrrk2 has further been speculated to participate in the pathophysiology of inflammatory bowel disease (IBD)^4^, leprosy^5^, and cancer^6^. Lrrk2 may be effective by regulating inflammatory processes^7^,^8^,^9^. Lrrk2 is expressed in several circulating leukocytes, such as CD14^+^ monocytes, CD19^+^B cells, CD4^+^T cells and CD8^+^T cells^10^. Lrrk2 is further expressed in dendritic cells (DCs)^10^,^11^, antigen-presenting cells linking innate and adaptive immunity and contributing to stimulation of regulatory T cell differentiation, which impacts on the maintenance of self-tolerance^12^,^13^,^14^,^15^.

Lrrk2 contributes to signalling of interferon γ^11^,^16^, NF-κB-dependent transcription^11^ and regulation of reactive oxygen species (ROS) production^11^. Lrrk2 is up-regulated by bacterial lipopolysaccharide and lentiviral particles^10^ and contributes to monocyte maturation^17^. Lrrk2 participates in the regulation of microglia inflammation and neurodegeneration^18^. However, cellular mechanisms accounting for Lrrk2 dependent pathophysiology of inflammation and PD are still incompletely understood.

Lrrk2 is involved in Ca^2+^ signaling^19^. According to recent observations Lrrk2 up-regulates Na^+^/Ca^2+^ -exchanger expression and activity in DCs thus blunting Ca^2+^ -signals and attenuating Ca^2+^ -dependent functions of DCs^20^. Upregulation of Na^+^/Ca^2+^ -exchanger expression and activity could decrease cytosolic Ca^2+^ activity ([Ca^2+^]i) only, if the electrochemical Na^+^ gradient is high enough to extrude Ca^2+^ against its steep electrochemical gradient. Na^+^/Ca^2+^ -exchanger function thus requires maintenance of the Na^+^ gradient across the cell membrane, a function of the Na^+^/K^+^ ATPase^21^. Inhibition of the Na^+^/K^+^ ATPase dissipates the Na^+^ gradient across the cell membrane, leads to reversal of the driving force of Na^+^/Ca^2+^ -exchange and thus increases [Ca^2+^]i^22^,^23^. To the extent that a function of Lrrk2 is the stimulation of Ca^2+^ extrusion by up-regulation of Na^+^/Ca^2+^ -exchangers, the efficacy of the kinase requires adequate Na^+^/K^+^ ATPase activity. We hypothesized that Lrrk2 may, in addition to its effect on Na^+^/Ca^2+^ -exchange, up-regulate Na^+^/K^+^ ATPase activity.

The present study thus explored whether Lrrk2 participates in the regulation of Na^+^/K^+^ ATPase activity in DCs. DCs were isolated from gene-targeted mice lacking Lrrk2 (*Lrrk2*^−/−^) and their wild-type littermates (*Lrrk2*^+/+^) and Na^+^/K^+^ ATPase expression at mRNA and protein levels determined by RT-PCR and Western blotting, respectively. Na^+^/K^+^ ATPase activity was measured by patch clamp. As a result, Na^+^/K^+^ ATPase expression and activity were indeed lower in *Lrrk2*^−/−^ DCs than in *Lrrk2*^+/+^ DCs. Moreover, treatment of *Lrrk2*^+/+^ DCs with Lrrk2 inhibitor GSK2578215A decreased Na^+^/K^+^ ATPase activity. The up-regulation of Na^+^/K^+^ ATPase activity contributes to the maintenance of the steep electrochemical Na^+^ gradient required for Ca^2+^ extrusion by the Na^+^/Ca^2+^ -exchanger.

## Results

The present study explored, whether Lrrk2 has an impact on the Na^+^/K^+^ -ATPase activity in DCs. To this end ouabain sensitive K^+^ -induced outward currents were recorded utilizing whole cell patch clamp in DCs isolated from bone marrow of gene-targeted mice. Comparison was made between DCs isolated from mice lacking functional Lrrk2 (*Lrrk2*^−/−^) and DCs isolated from their wild type littermates (*Lrrk2*^+/+^).

**Figure 1:**
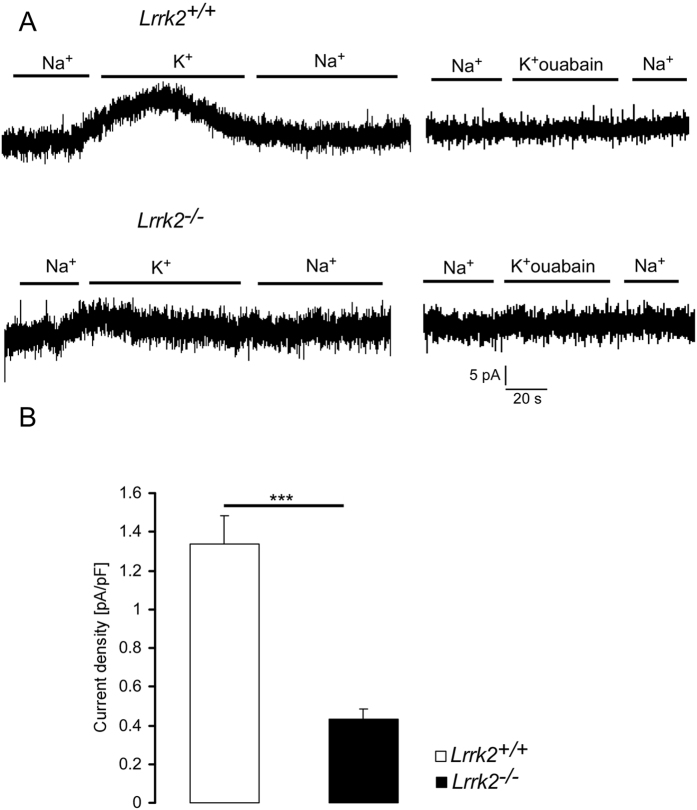
Na^+^/K^+^ -ATPase currents in *Lrrk2*^−/−^ and *Lrrk2*^+/+^ DCs. (**A**) Original whole cell recordings at −40 mV in *Lrrk2*^+/+^ DCs (upper) and *Lrrk2*^−/−^ DCs (lower) in absence (Na^+^) and presence of 5 mM K^+^ (K^+^) in bath. K^+^ was added in absence (left) or presence (right) of ouabain (100 μM). (**B**) Means ± SEM (n = 7-8) of whole-cell current at −40 mV normalized to cell capacitance in *Lrrk2*^+/+^ DCs (white bar) and *Lrrk2*^−/−^ DCs (black bar). ***(p < 0.001) indicates significant difference between genotypes, unpaired t-test.

As shown in Fig. 1, the addition of 5 mM K^+^ to the bath solution was followed by an outward current, which was significantly smaller in DCs from *Lrrk2*^−/−^ mice than in DCs from *Lrrk2*^+/+^mice. In both genotypes, the K^+^ induced current was abrogated by the addition of 100 μM ouabain (Fig. 1A,B).

**Figure 2:**
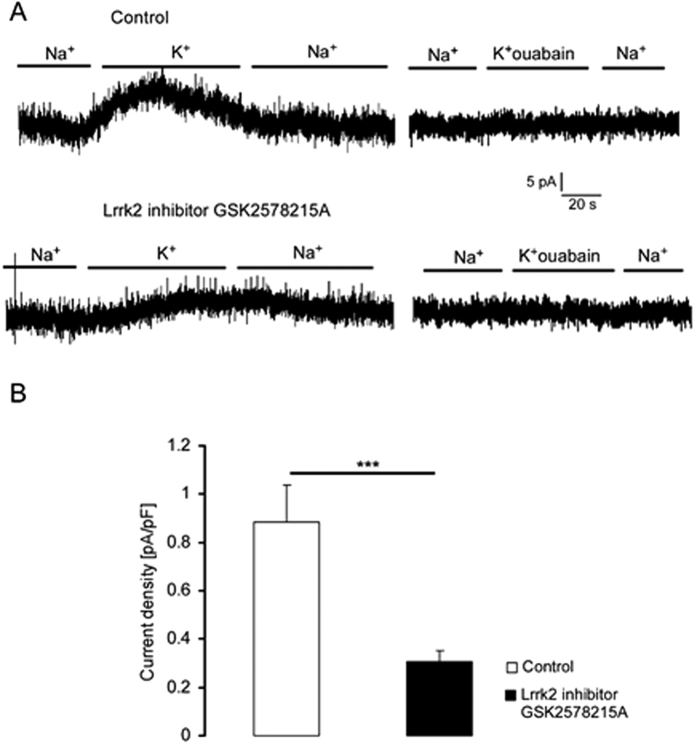
Sensitivity of Na^+^/K^+^ -ATPase currents in *Lrrk2*^+/+^ DCs to Lrrk2-inhibitor GSK2578215A. (**A**) Original whole cell recordings at −40 mV in *Lrrk2*^+/+^ DCs in absence (Na^+^) and presence of 5 mM K^+^ (K^+^) in bath prior to (upper) and following (lower) 24 hours treatment with Lrrk2-inhibitor GSK2578215A (1 μM) in absence (left) or presence (right) of ouabain (100 μM). (**B**) Means ± SEM (n = 5–10) of whole-cell current at −40 mV normalized to cell capacitance in *Lrrk2*^+/+^ DCs without (control; white bar) and with (1 μM; black bar) a prior 24 hours exposure to Lrrk2-inhibitor GSK2578215A. ***(p < 0.001) indicates significant difference from absence of inhibitor, unpaired t-test.

Further experiments explored whether genetic knockout of Lrrk2 was mimicked by pharmacological inhibition of the kinase by the Lrrk2 inhibitor GSK2578215A. As shown in Fig. 2, a 24 hours pre-treatment of *Lrrk2*^+/+^ DCs with GSK2578215A (1 μM, 24 hours) was followed by a significant decrease of K^+^ induced current. In both, the presence and absence of GSK2578215A, the K^+^ induced current was abrogated by the addition of 100 μM ouabain (Fig. 2A,B).

**Figure 3:**
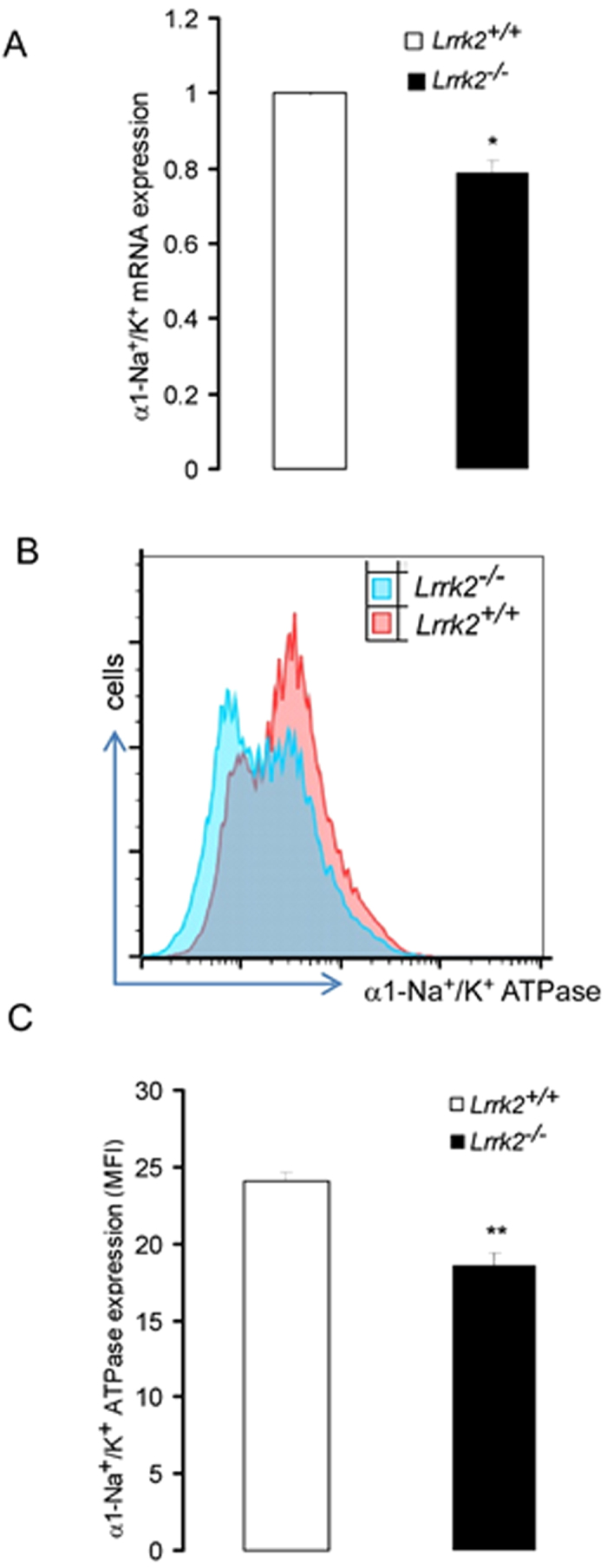
Lrrk2-dependent regulation of Na^+^/K^+^ -ATPase α1-subunit transcript and protein levels in *Lrrk2*^−/−^DCs and *Lrrk2*^+/+^ DCs. (**A**) Means ± SEM (n = 3) of α1-subunit Na^+^/K^+^ -ATPase transcript levels in *Lrrk2*^+/+^ DCs (white bar) and *Lrrk2*^−/−^ DCs (black bar) with Gapdh as reference (*Lrrk2*^−/−^DCs values normalized to *Lrrk2*^+/+^ DCs). (**B**) Original histogram of Na^+^/K^+^ -ATPase protein abundance determined by flow cytometry in *Lrrk2*^+/+^ DCs (red) and *Lrrk2*^−/−^ DCs (blue). (**C**) Means ± SEM (n = 4) of Na^+^/K^+^ -ATPase protein abundance (mean fluorescence intensity; MFI) determined by flow cytometry in *Lrrk2*^+/+^ DCs (white) and *Lrrk2*^−/−^ DCs (black). *(p < 0.05), **(p < 0.01) indicates significant difference between genotypes, unpaired t-test.

In order to test whether Lrrk2 influences Na^+^/K^+^ -ATPase at the transcript and/or protein level, transcript levels of the Na^+^/K^+^ -ATPase α1 subunit were analyzed by RT-PCR and protein expression was analyzed using flow cytometry. As illustrated in Fig. 3A. the transcript levels of the Na^+^/K^+^ -ATPase α1 subunit were significantly lower in *Lrrk2*^−/−^ DCs than in *Lrrk2*^+/+^ DCs. Thus, in the absence of Lrrk2 Na^+^/K^+^ -ATPase transcript levels in DCs are reduced. Decreased transcript levels were accompanied by a reduction of Na^+^/K^+^ -ATPase α1 subunit protein levels. Analysis using flow cytometry revealed that Na^+^/K^+^ -ATPase α1 subunit protein abundance was lower in *Lrrk2*^−/−^ DCs as compared to *Lrrk2*^+/+^ DCs (Fig. 3B,C).

**Figure 4:**
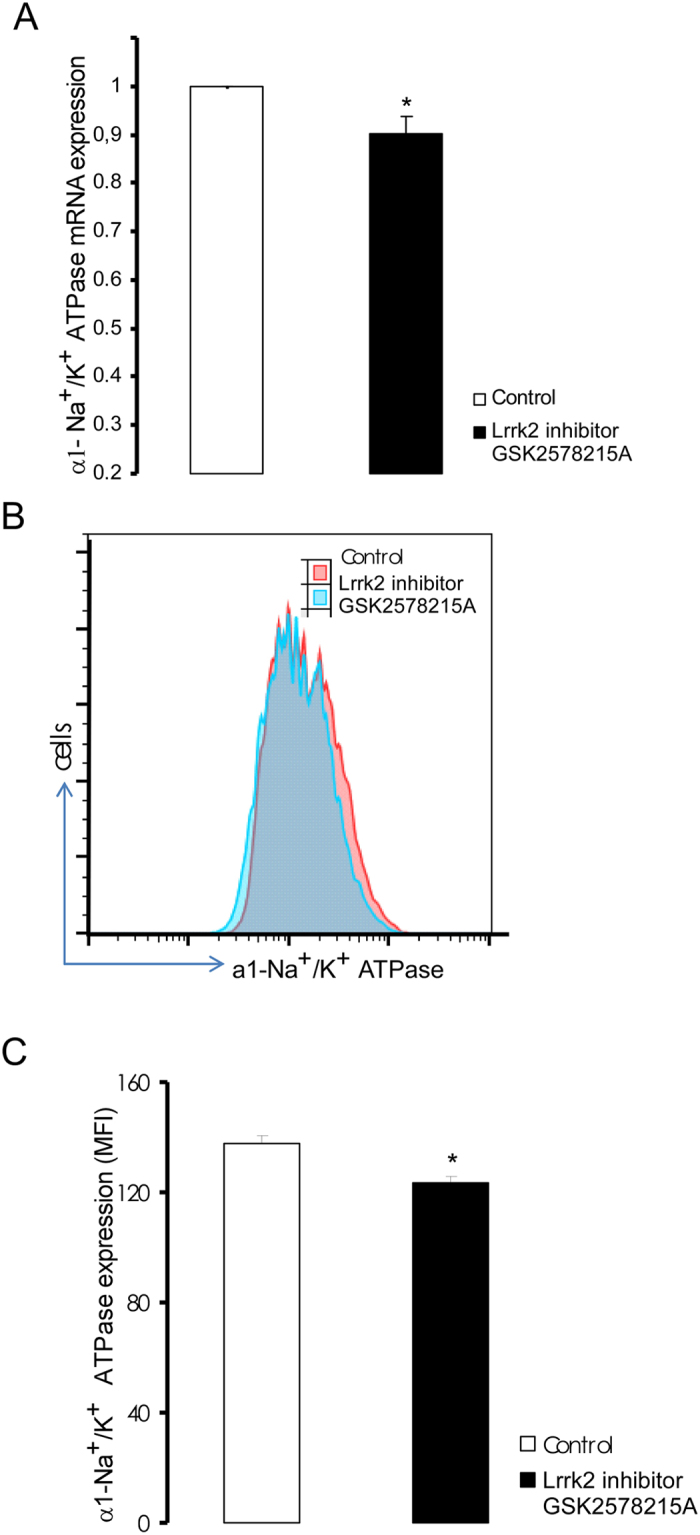
Lrrk2-dependent Na^+^/K^+^ -ATPase α1-subunit transcript and protein levels in *Lrrk2*^+/+^ DCs without or with prior pharmacological inhibition of Lrrk2. (**A**) Means ± SEM (n = 3) of mRNA encoding Na^+^/K^+^ -ATPase α1-subunit in *Lrrk2*^+/+^ DCs without (white) and with (black) prior exposure to the Lrrk2 inhibitor GSK2578215A (1 μM, 24 hours) with Gapdh as reference (treated normalized to respective untreated *Lrrk2*^+/+^ DCs). (**B**) Original histogram of Na^+^/K^+^ -ATPase protein abundance quantified by flow cytometry in untreated (red) and GSK2578215A (1 μM, 24 hours) treated (blue) *Lrrk2*^+/+^ DCs. (**D**) Means ± SEM (n = 3) of Na^+^/K^+^ -ATPase protein abundance (mean fluorescence intensity; MFI) in untreated (white) and GSK2578215A (1 μM, 24 hours) treated (black) *Lrrk2*^+/+^ DCs. *(p < 0.05) indicates significant difference from absence of inhibitor, unpaired t-test.

Additional experiments explored whether pharmacological inhibition of the kinase by the Lrrk2 inhibitor GSK2578215A influences Na^+^/K^+^ -ATPase α1 subunit expression. As illustrated in Fig. 4A, a 24 h treatment of *Lrrk2*^+/+^ DCs with GSK2578215A (1 μM, 24 hours) resulted in a significant decrease of Na^+^/K^+^ -ATPase α1 subunit transcript levels and protein abundance of the Na^+^/K^+^ -ATPase α1 subunit (Fig. 4B,C).

## Discussion

The present observations demonstrate that Lrrk2 affects expression levels and activity of the Na^+^/K^+^ -ATPase in DCs. We show that Na^+^/K^+^ -ATPase activity is lower in DCs isolated from mice lacking functional Lrrk2 (*Lrrk2*^−/−^) as compared to DCs isolated from wild type littermates (*Lrrk2*^+/+^). The down-regulation of Na^+^/K^+^ -ATPase activity in Lrrk2 deficient dendritic cells (DCs) is accompanied by a decrease in the level of Na^+^/K^+^ -ATPase α1 subunit-encoding transcripts and of the Na^+^/K^+^ -ATPase α1 subunit membrane protein.

The present observations do not allow safe conclusions concerning the mechanisms accounting for the decrease of Na^+^/K^+^ -ATPase α1 subunit expression in Lrrk2 deficient DCs. However, it is noteworthy that Lrrk2 influences the activity of several transcription factors. Lrrk2 up-regulates the activity of nuclear factor κB (NF-κB) by stimulation of expression and by phosphorylation of the inhibitor IκBα^11^,^24^,^25^. NF-κB has in turn been shown to up-regulate the Na^+^/K^+^ -ATPase^26^. Lrrk2 further up-regulates the forkhead box transcription factor FoxO1 by direct phosphorylation^27^. Lrrk2 retains the transcription factor nuclear factor of activated T cells (NFAT) in the cytoplasma and Lrrk2 deficiency leads to nuclear up-regulation of NFAT^28^,^29^.

A comparison of Na^+^/K^+^ -ATPase α1 subunit expression (Figs 3 and 4) and Na^+^/K^+^ -ATPase activity (Figs 1 and 2) suggests that Na^+^/K^+^ -ATPase α1 subunit expression does not fully account for the differences in Na^+^/K^+^ -ATPase activity. Thus, Lrrk2 may, in addition to its effect on expression, modify the activity of expressed Na^+^/K^+^ -ATPase protein. In theory, Lrrk2 may directly phosphorylate the pump protein or may influence signalling molecules regulating Na^+^/K^+^ -ATPase activity. It is noteworthy that Lrrk2 activates the protein kinase B (PKB/Akt)^30^, which shares the consensus sequence with serum and glucocorticoid inducible kinase SGK1^31^, a kinase known to up-regulate Na^+^/K^+^ ATPase^32^.

The finding that Na^+^/K^+^ ATPase activity is significantly lower in *Lrrk2*^−/−^ DCs than in *Lrrk2*^+/+^ DCs under baseline conditions suggests that Lrrk2 constitutively controls Na^+^/K^+^ ATPase activity. This is strongly supported by pharmacological inhibition of Lrrk2 in DCs. A 24 hours exposure of *Lrrk2*^+/+^ DCs to the Lrrk2 inhibitor GSK2578215A decreased Na^+^/K^+^ -ATPase expression and activity to a similar extent as the genetic knockout of the kinase in DCs. Collectively, these findings indicate that Lrrk2 kinase activity and expression in DCs accounts for the observed differences in DC Na^+^/K^+^ -ATPase activity

Downregulation of Na^+^/K^+^ -ATPase activity either by pharmacological agents^33^, a decrease in temperature^34^ or energy depletion^35^, can lead to inhibition of K^+^ channels and result in cellular depolarization and dissipation of the electrical driving force for Na^+^ coupled transport^36^. Carriers affected by compromised Na^+^/K^+^ -ATPase activity include the Na^+^/Ca^2+^ exchangers^37^,^38^, which were previously shown to be regulated in a Lrrk2-dependent fashion^20^. Notably, Lrrk2 also affects Ca^2+^ signaling in neurons^39^ and in these excitable cells, depolarization due to down-regulation of Na^+^/K^+^ ATPase may modify cytosolic Ca^2+^ activity by activation of voltage gated Ca^2+^ channels^40^,^41^. To which extent Lrrk2-dependent Na^+^/K^+^ -ATPase activity and the activity of Na^+^/Ca^2+^ exchangers are linked, remains to be shown. In DCs, Lrrk2 clearly impacts on cytosolic Ca^2+^ activity, which participates in the regulation of diverse DC functions^42^ including maturation, synthesis of inflammatory cytokines and induction of oxidative burst^39^.

Na^+^/K^+^ -ATPase activity also impacts on cellular energy metabolism. The Na^+^/K^+^ ATPase is responsible for a large fraction (20–80%) of metabolic rate^43^ and accounts for about 30% of cellular ATP consumption^44^,^45^,^46^,^47^. In hypoxic microenvironments, such as inflammatory or tumor tissues^48^ the ability to regulate the Na^+^/K^+^ ATPase could therefore be relevant for DC survival, function and DC-mediated immune responses.

Another consequence of decreased Na^+^/K^+^ -ATPase activity might include increased cytosolic Na^+^ levels and an induction of salt-inducible kinase 1, which is a powerful stimulator of Na^+^/K^+^ ATPase and is part of a negative feedback loop regulating the Na^+^/K^+^ -ATPase^49^. In brief, future studies are needed to unravel the likely complex contribution on DC cell function of pump regulation by Lrrk2.

In conclusion, the present study demonstrates for the first time a Lrrk2 sensitive regulation of Na^+^/K^+^ -ATPase expression and activity in bone marrow derived DCs. The impact of Lrrk2 on Na^+^/K^+^ -ATPase activity may affect multiple cellular functions in DCs and other cells and may be highly relevant in the pathophysiology of Lrrk2-pathway linked diseases.

## Materials and Methods

### Ethics Statement

All animal experiments were performed according to the German animal protection law and approved by the local authorities (Regierungspräsidium Tübingen).

### Mice

Dendritic cells (DCs) were isolated from gene targeted mice lacking functional Lrrk2 (*Lrrk2*^−/−^) and their wildtype littermates (*Lrrk2*^+/+^). Origin of the mice, breeding and genotyping were described previously^50^. Male and female mice were studied at the age of 8–12 weeks. The mice had access to water ad libitum and to standard food (Altromin 1310).

### Cell Culture

Dendritic cells (DCs) were cultured from bone marrow of 8–12 weeks old female and male *Lrrk2*^+/+^and *Lrrk2*^−/−^ mice. Bone marrow derived cells were flushed out of the cavities from the femur and tibia with PBS^51^. Cells were then washed twice with RPMI and seeded out at a density of 2 × 10^6^ cells per 60-mm dish. Cells were cultured for 7 days in RPMI 1640 with L-Glutamine (GIBCO, Carlsbad, Germany) containing: 10% FCS, 1% penicillin/streptomycin, 1% non-essential amino acids (NEAA) and 0.05% β-mercaptoethanol. Cultures were supplemented with GM-CSF (35 ng/mL, Immunotools, Germany) and fed with fresh medium containing GM-CSF on days 3 and 6. At day 7, >95% of the cells expressed CD11c, which is a marker for mouse DCs. Experiments were performed on DCs at days 7–9.

### Flow cytometry

Bone marrow derived DCs from *Lrrk2*^−/−^ and *Lrrk2*^+/+^mice were characterised by using surface and intracellular staining with anti-Mouse CD11c-APC (eBiosciences; clone N418), anti-Mouse MHCII-PE (BD Biosciences; M5/114.15.2), rabbit anti-mouse-Na^+^/K^+^ ATPase α1 subunit protein (Cell Signaling, USA) and Goat anti-Rabbit IgG-FITC (Santa Cruz Biotech, USA; sc-2012). To characterise the DCs, 200 × 10^3^ BMDCs were collected and centrifuged at 600 *g* for 5 minutes at room temperature and washed once with 1x DPBS (Sigma, Germany). 0.5 μl of antibody containing solution (0.2 μg/μl anti-CD11c-APC and anti-MHC II-PE) were added to 50 μl of DPBS and cells were incubated for 30 minutes at room temperature in the dark. After incubation, cells were washed once with DPBS and fixed with 100 μl of fixation/permeabilization buffer (eBioscience, Germany) for 30 minutes in the dark and washed once with 1x permeabilization buffer (eBioscience, Germany). After washing, 0.5 μl antibody containing solution (1.0 μg/μl anti-mouse-Na^+^/K^+^ ATPase α1) was added to 50 μl permeabilization buffer, cells were incubated in the dark for 45 minutes and cells were washed twice with 1x permeabilization buffer. After washing 0.2 μl Goat anti-Rabbit IgG-FITC in 50 μl of 1x permeabilization buffer was added and incubated for another 30 minutes in the dark. Finally, the cells were washed twice with DPBS and added 200 μl of DPBS. All washing steps were performed at 600 *g* for 5 minutes and room temperature. Cells were acquired using BD FACSCalibur™ (BD Bioscience, Heidelberg, Germany) flow cytometry and data were analysed by Flowjo (Treestar, USA)^53^. CD11c^+^ DCs were gated for Na^+^/K^+^ ATPase α1 protein expression, which is presented in mean fluorescence intensity (MFI).

### Real-time PCR

Total RNA was extracted from mouse dendritic cells in PureLink™ RNA Mini Kit (Life Technologies, Germany) according to the manufacturer’s instructions^54^. Total RNA was used for cDNA synthesis using Superscript III cDNA Synthesis kit (Life technologies, Germany) according to the manufacturer’s instructions. Polymerase chain reaction (PCR) amplification of the respective genes were set up in a total volume of 10 μl using 10 ng of cDNA, 250 nM forward and reverse primer and 2x qPCR Master Mix KAPA SYBR Green (PeqLab, Erlangen, Germany) according to the manufacturer’s protocol. Cycling conditions were used as follows: initial denaturation at 95 °C for 3 min, followed by 40 cycles of 95 °C for 10 sec, 60 °C for 1 min and then melting curve analysis protocol was performed. For the amplification the following primers were used (5′->3′orientation): Atp1α1 F: AGCATCAATGCGGAGGATGT, R: TATCCACCTTGCAGCCGTTT and Gapdh; F: CGT CCC GTA GAC AAA ATG GT; R: TTG ATG GCA ACA ATC TCC AC.

Specificity of PCR products was confirmed by analysis of melting curves. Real-time PCR amplifications were performed on a CFX96 Real-Time System (Bio-Rad). All experiments were done in duplicate. Amplification of the house-keeping gene GAPDH was performed to standardize the amount of sample RNA. Relative quantification of gene expression was achieved using the ^ΔΔ^ct method as described earlier^55^.

### Patch clamp

Ouabain-sensitive K^+^ -induced currents (I_pump_) reflecting Na^+^/K^+^ -ATPase activity were determined by whole cell patch clamp recording in *Lrrk2*^−/−^ and *Lrrk2*^+/+^ DCs as well as in *Lrrk2*^+/+^ DCs in absence and presence of the LRRK2 inhibitor GSK2578215A (1 μM, 24 hours) (Tocris, United Kingdom). Whole cell patch clamp experiments were performed at room temperature in voltage-clamp, fast whole cell mode^56^. Cells were continuously superfused through a flow system inserted into the dish. The bath was grounded via a bridge filled with the external solution. Borosilicate glass pipettes (2- to 4-MΩ resistance; Harvard Apparatus, UK) manufactured by a microprocessor-driven DMZ puller (Zeitz, Augsburg, Germany), were used in combination with a MS314 electrical micromanipulator (MW, Märzhäuser, Wetzlar, Germany). The currents were recorded by an EPC-9 amplifier (Heka, Lambrecht, Germany) and analyzed with Pulse software (Heka) and an ITC-16 Interface (Instrutech, Port Washington, NY). Currents were recorded at an acquisition frequency of 10 kHz and 3 kHz low-pass filtered^57^. To measure Na^+^/K^+^ ATPase activity, ouabain (100 μM) sensitive K^+^ -induced outward currents were recorded^53^. The pipette solution contained (in mM): 30 NaCl, 20 KCl, 70 CsCl, 5 MgCl_2_, 5 HEPES, 5 Na_2_ATP and 5 ethylene glycol tetraacetic acid (EGTA). The external solution contained (in mM) 60 NaCl, 80 TEA-Cl, 1 MgCl_2_, 2.5 CaCl_2_, 5 NiCl_2_, 5 glucose, 10 HEPES (pH 7.4, CsOH), and 0.5 EGTA. Na^+^/K^+^ ATPase currents were elicited by switching to a bath solution that contained 60 NaCl, 80 TEA-Cl, 5 KCl, 1 MgCl_2_, 2.5 CaCl_2_, 5 NiCl_2_, 5 glucose, 10 HEPES (pH 7.4, CsOH). The currents were measured at −40 mV.

### Statistical analysis

Data are provided as means ± SEM, n represents the number of independent experiments. Data were tested for significance using unpaired student´s t-test. Results with p < 0.05 were considered statistically significant.

## Additional Information

**How to cite this article**: Hosseinzadeh, Z. *et al*. Leucine-Rich Repeat Kinase 2 (Lrrk2)-Sensitive Na^+^/K^+^ ATPase Activity in Dendritic Cells. *Sci. Rep.* **7**, 41117; doi: 10.1038/srep41117 (2017).

**Publisher's note:** Springer Nature remains neutral with regard to jurisdictional claims in published maps and institutional affiliations.
